# Intermittent Blurry Vision: An Unexpected Presentation of Cushing’s Syndrome Due to Primary Bilateral Macronodular Adrenal Hyperplasia (PBMAH)

**DOI:** 10.7759/cureus.25017

**Published:** 2022-05-15

**Authors:** Christopher Fernandez, Smriti Bhatia, Ariana Rucker, Lee Celio

**Affiliations:** 1 Internal Medicine, Nazareth Hospital, Philadelphia, USA; 2 Internal Medicine, Philadelphia College of Osteopathic Medicine (PCOM), Philadelphia, USA

**Keywords:** macronodular, adrenal hyperplasia, bilateral, acth-independent, cushing's syndrome

## Abstract

Cushing’s syndrome (CS) is an uncommon endocrine disorder resulting from prolonged exposure to elevated glucocorticoids, with 10-15 million annual cases per the American Association of Neurological Surgeons. Exogenous and endogenous causes can further be divided into adrenocorticotropic hormone (ACTH) dependent (i.e Cushing’s Disease) or ACTH independent. ACTH-independent CS can be caused by primary bilateral macronodular adrenal hyperplasia (PBMAH) representing less than 1% cases of CS. We report a case of a woman presenting with chronic resistant hypertension, episodic blurry vision, weight gain and wasting of extremities. She was diagnosed with Cushing’s syndrome due to PBMAH.

Our patient’s presentation was unusual as she presented at 40 years old, 10 years earlier than expected for PBMAH; and primarily with complaints of episodic blurry vision. Her symptoms also progressed rapidly as signs and symptoms largely presented over the course of 12 months, however responded well to surgical resection.

## Introduction

Cushing’s syndrome (CS) is an uncommon endocrine disorder caused by prolonged exposure to elevated glucocorticoids [1]. There are exogenous or endogenous causes. The National Institute of Health’s (NIH) Genetic and Rare Diseases Information Center (GARD) estimated the prevalence of endogenous CS to be 1 in 26,000 [2]. According to a large study, the annual incidence of CS in individuals less than 65 years old was nearly 49 cases per million [3]. Cushing’s disease (CD), which is defined as Cushing’s syndrome caused by an adrenocorticotropic hormone (ACTH)-secreting pituitary tumor, accounts for approximately 80% of patients with CS; whereas ACTH-independent CS accounts for the remaining 20% [4]. Among the causes of pituitary ACTH-independent CS is bilateral macronodular adrenal hyperplasia which is rare, comprising less than 1% of patients with CS [5]. Herein is a case of rapid onset Cushing’s syndrome due to PBMAH initially presenting as episodes of bilateral blurry vision.

## Case presentation

The patient is a 40-year-old female with a past medical history of resistant hypertension (on four agents), and recently diagnosed type 2 diabetes mellitus (started on insulin regimen). Patient was recently seen by her primary care provider, with complaints of intermittent episodes of blurry vision going on for months. 

As part of evaluation in December 2020, the patient underwent a renal ultrasound as part of evaluation by the primary physician for uncontrolled hypertension. The doppler incidentally showed an indeterminate hypoechoic mass on the right kidney and presumably located within the right adrenal gland, measuring 3.4 x 5.4 cm, without sonographic evidence of renal artery stenosis. The left kidney appeared normal. She was recommended to have further evaluation with contrast enhanced MR or CT with adrenal protocol.

**Figure 1:**
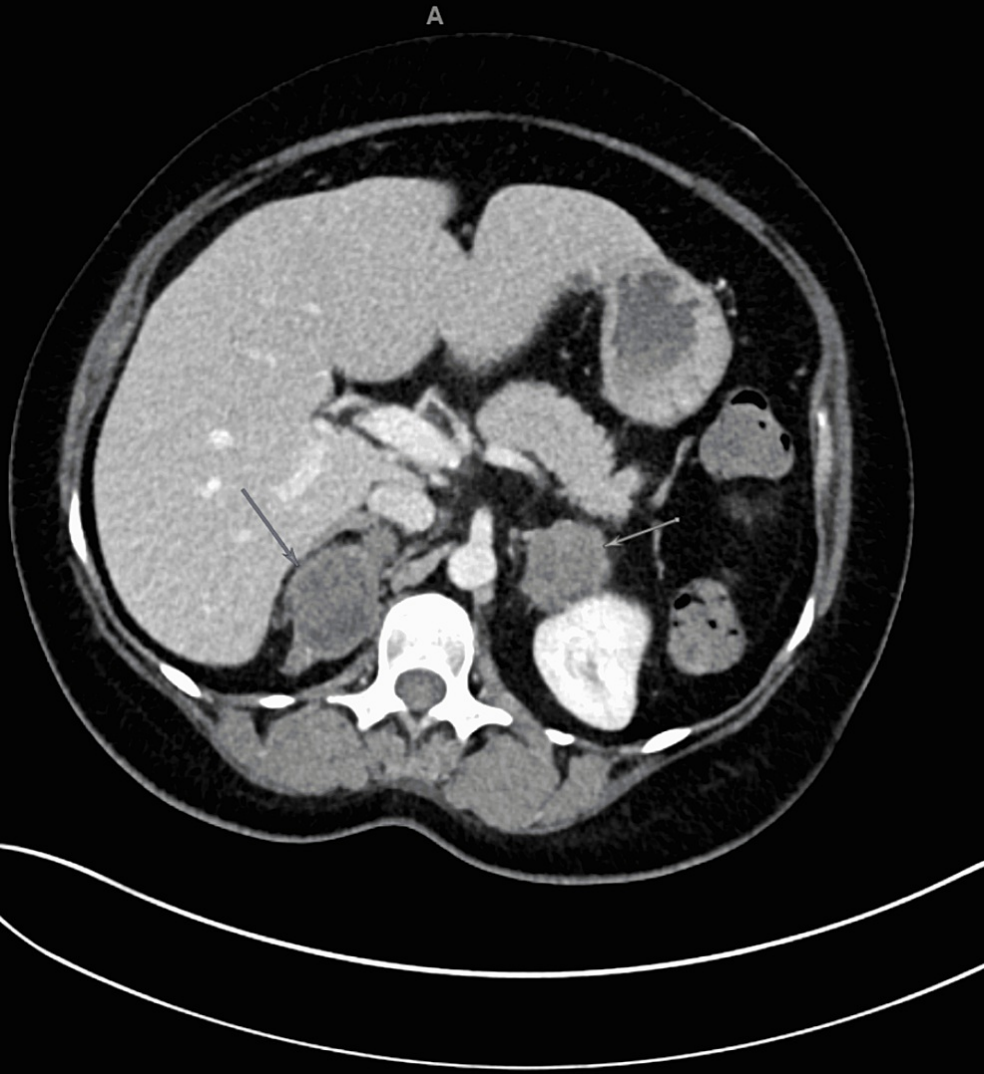
CT of the abdomen/pelvis with contrast showing low-attenuation masses present in both adrenal glands measuring 6.9 x 5.3 cm on the right (dark gray arrow) and 4.5 x 3.9 cm on the left (light gray arrow)

In January 2021, the patient was sent from her PCP’s office to the ED as the patient was having blurred vision. She had a plain CT scan of the brain that was unremarkable. The patient's systolic blood pressure was in the 160s-170s mm Hg upon arrival to ED compliance with home medications of 5mg of amlodipine daily, 25mg of metoprolol succinate daily, 100mg of losartan daily, and 25mg of hydrochlorothiazide daily. Physical exam reported obesity without evidence of abdominal striae. Blood work in the ED showed elevated blood glucose level over 600 (mg/dL) despite being on a regimen of lantus 60 units, metformin 1000mg twice a day, and semaglutide SQ weekly. Hemoglobin A1c was greater than 15.5%, and vitamin D was low (15.6 ng/mL). The morning ACTH was low (<5pg/mL) (nAM levels: 7.2 - 63.3 pg/mL), AM cortisol was high at 26.1 ug/ml (normal: 5.0 - 23.0 ug/mL), plasma aldosterone was normal at 4.2 ng/dL with a normal plasma renin at 1.96 (0.25 - 5.82 ng/mL/h). 24-hour urine free cortisol (UFC) was high at 1299.5 (4.0-50.0 mcg/24h). CT of the abdomen/pelvis with and without contrast showed low-attenuation masses (less than 5 Hounsfield units) present in both adrenal glands measuring 6.9 x 5.3 cm on the right and 4.5 x 3.9 cm on the left, and did not demonstrate significant arterial enhancement (Figure 1). MR imaging of the abdomen without and with contrast was also obtained and showed the same masses of the bilateral adrenal glands, with largest on the left measured 3.6 cm and largest on the right measured 3.7 cm, as well as mild fatty infiltration of the liver. General surgery and hematology/oncology were consulted and recommendations were made for outpatient follow-up with PCP and endocrinology. 

In early February 2021, the patient again presented to the ED complaining of recurrent episodes of bilateral blurry vision. Examination was unremarkable, including an ophthalmological exam with slit lamp exam. Blurred vision was suspected to be due to osmotic swelling in the setting of severe hyperglycemia as the patient had persistently uncontrolled blood sugars. Recommendations were for tighter control of blood glucose, and follow-up with primary care and ophthalmology. 

Patient followed up with the endocrinologist in mid-February to which the patient reported first noticing a difference in her energy and changes to her weight around one year prior. She communicated a weight gain of 30 to 40 lbs over the past year. Patient had a reported history of gestational hypertension diagnosed five years ago when she gave birth to her daughter, which was steadily worsening over the past year. She reported intermittent myalgias and easy bruising. Patient had no family history or any apparent features to suggest multiple endocrine neoplasia (MEN) syndrome. Blood work revealed ACTH less than 1.5 pg/mL, AM cortisol was high at 24.5 mcg/dL, and normal aldosterone at 3.6 ng/dL, with normal renin and metanephrine levels. Physical examination revealed truncal obesity as well as a round face, cushingoid in appearance, and relatively thin extremities and abdominal striae.

She was then referred to a surgical specialist, and it was decided that she would undergo laparoscopic bilateral adrenalectomy due to severe Cushing’s syndrome. The surgical pathology report revealed macro-nodular cortical hyperplasia of both left and right adrenal gland masses with random endocrine atypia. The largest nodule on the left measured 4.5 cm and the largest nodule on the right measured 6.6 cm. Post-operatively she was started on hydrocortisone 20 mg every morning and 10 mg every evening, and fludrocortisone 0.1 mg twice a day as part of her steroid replacement regimen. Eventually she changed to hydrocortisone 10 mg three times a day and fludrocortisone 0.1 mg once a day. For her diabetes, her insulin glargine decreased from 60 units to 20 units. Amlodipine and hydrochlorothiazide were discontinued from her antihypertensive medications; she continued losartan and metoprolol. Follow up blood work showed stable electrolytes with potassium 4.2 mmol/L (3.5-5.2 mmol/L), sodium 137 mmol/L (134-144mmol/L), chloride 100 mmol/L (96-106 mmol/L), and carbon dioxide 23 mmol/L (20-29mmol/L).

## Discussion

ACTH-independent Cushing’s syndrome due to bilateral cortisol-secreting nodules is rare, accounting for 2% of CS cases. The majority of causes include primary bilateral macronodular adrenal hyperplasia (PBMAH), primary pigmented nodular adrenocortical disease (PPNAD), and bilateral adrenocortical adenomas (BAA). In PBMAH, typically patients are diagnosed within the fifth or sixth decade of life [4]. The usual age of onset for PPNAD is within the first to third decade of life, with median age in the pediatric population at age 15 years [6]. BAA is such a rare entity that there exists little epidemiological data with less than 40 reported cases until 2019 [7]. A small subset of patients present with overt clinical symptoms of CS, as hypercortisolism often follows an insidious course that can delay diagnosis from years to decades, with one series reporting a diagnostic delay of approximately eight years [8]. Serum and urine hormone screening in the right clinical setting can provide clues to these endocrine disorders, however diagnosis of ACTH-independent CS often occurs incidentally wherein a radiographic study was done for reasons other than to identify adrenal disease [9]. CT or MRI alone are not able to differentiate these disease entities, requiring pathological examination for final determination [7]. Adrenal venous sampling (AVS) and I-6B-iodomethyl-19-norcholesterol (I-NP-59) can aid in identifying hormone-secreting status of each adrenal lesion, however usefulness is debated among experts [10-12].

In all cases the end goal is to normalize adrenocortical hormones, and PBMAH primarily involves surgical resection with exogenous hormone replacement. Bilateral adrenalectomy is generally the treatment of choice with overt Cushing syndrome regardless of cortisol level. These patients require lifelong steroid administration [9,13]. Another approach is unilateral adrenalectomy of the larger or more metabolically active gland, which can be identified after AVS or I-NP-59 testing. This has been proposed in order to preserve some autonomous hormonal production and prevent adrenal crisis, however remission rates of Cushing syndrome as high as 84% have been reported with eventual need for bilateral adrenalectomy [7,8,14]. Steroid enzyme inhibition to control cortisol secretion has been used as an adjunct before surgery. In some patients with identified aberrant adrenal hormone receptors, targeted pharmacological inhibition remains an alternative medical approach [8]. Despite these alternatives to surgery, surgical resection remains the optimal approach [1].

## Conclusions

ACTH-independent Cushing’s syndrome due to PBMAH usually presents as an indolent course, with typical diagnosis in the fifth to sixth decade. As the use of imaging for other non-endocrine related investigations becomes more utilized, PBMAH being less of a rare entity. Clinical presentation usually dictates the timing of and type of surgical intervention. Although there are some reports of unilateral resection resulting in a cure, many of these cases eventually proceed to staged bilateral resection. Our patient’s presentation as her primary complaint was recurrent episodes of blurry vision that were suspected to be due to osmotic swelling because of her uncontrolled hyperglycemia. Her case was also unusual as she presented at 40 years old, an average of 10 years earlier than is typically diagnosed for PBMAH. Her symptoms also progressed rapidly over the course of 12 months with development of resistant hypertension and insulin-dependent diabetes requiring high basal insulin. Following surgical resection, her antihypertensive regimen was de-escalated and had significant reduction in insulin requirements, and was maintained on adrenocorticoid therapy.
